# Human BK Polyomavirus Plasmid pBKV (34-2) (Dunlop) Contains Mutations Not Found in the Originally Published Sequences

**DOI:** 10.1128/genomeA.00046-15

**Published:** 2015-03-26

**Authors:** Stian Henriksen, Christian Mittelholzer, Rainer Gosert, Hans H. Hirsch, Christine Hanssen Rinaldo

**Affiliations:** a Department of Microbiology and Infection Control, University Hospital of North Norway, Tromsø, Norway; b Transplantation and Clinical Virology, Department Biomedicine (Haus Petersplatz), University of Basel, Basel, Switzerland; c Infectious Diseases and Hospital Epidemiology, University Hospital Basel, Basel, Switzerland; d Metabolic and Renal Research Group, UiT The Arctic University of Norway, Tromsø, Norway

## Abstract

The plasmid pBKV (34-2) (ATCC 45025) contains the entire BK polyomavirus Dunlop genome. Sequencing revealed 12 point mutations compared to the GenBank sequence, but only 4 point mutations compared to the published sequence. The origin of these differences is unknown, but may impact virological as well as diagnostic research and development.

## GENOME ANNOUNCEMENT

The human BK polyomavirus (BKPyV) was first isolated in 1970 from the urine of a kidney transplant patient with ureteric stenosis shedding decoy cells (1). The isolate was passaged in cell culture and named after the senior author Susan Gardner and henceforth called the Gardner strain (2). In 1979, the whole-genome sequence of a derivative strain called Dunlop was reported (3). The circular double-stranded genome of 5,153 bp is divided into an early viral gene region (EVGR) encoding the regulatory proteins large, small, and truncated tumor antigen (LTag, sTag, and truncTag); a late viral gene region (LVGR) encoding the agnoprotein and the structural proteins VP1, VP2, and VP3; and a noncoding control region (NCCR) harboring the origin of replication and promoter-enhancer elements (4). The NCCR of BKPyV commonly found in urine is of a linear archetype architecture arbitrarily termed O_142_-P_68_-Q_39_-R_63_-S_63_, where the subscript indicates the respective length in base pairs. Rearrangements of these blocks frequently arise in cell culture (5–7) and in patients (8, 9), giving rise to new viral strain variants. In contrast, the EVGR and the LVGR are rarely affected. Although the origin of Dunlop is not clear, the NCCR is identical to the one of the Gardner strain, except for one additional 44-bp deletion removing the Q-block (3, 10). The Dunlop genome is available as a plasmid, pBKV (34-2) (ATCC 45025), which is frequently used for research and diagnostic purposes (10–22). ATCC refers to the GenBank number J02038.1, which has been replaced by the number V01108 referring to the original publication (3). Since the backbone of pBKV (34-2) is the low-copy vector pBR322, a high-copy plasmid was generated by cloning of the BKPyV genome into a pGEM vector (22). Dye-terminator cycle sequencing of the plasmid using 20 primers in the BKPyV genome and M13R and T7 primers in the vector (23) revealed 12 point mutations compared to the GenBank sequence, causing 6 amino acid (aa) substitutions affecting the VP2 gene (aa 100 Lys to Arg and aa 103 Asp to Ser), the VP1 gene (aa 158 Glu to Asp, aa 171 Ser to Thr, and aa 219 Ala to Thr) and the LTag gene (aa 260 Ser to Asn). However, compared to the originally published sequence (3), only 4 point mutations were found, resulting in 2 amino acid substitutions affecting the VP1 gene (aa 219 Ala to Thr) and the LTag gene (aa 260 Ser to Asn).

Sequencing of the original plasmid from ATCC gave identical results. The indicated changes in pBKV (34-2) may have accumulated over time, or alternatively, sequencing errors were already made during the original work. Since many laboratories use pBKV (34-2) for research and development, including as a positive control for real-time quantitative PCR (22), and since the GenBank sequence is frequently used to design primers and probes (22), the mutations detected may have consequences. The effect of these rather conservative amino acid substitutions on viral protein function is not known.

### Nucleotide sequence accession number.

The complete genome sequence of pBKV (34-2) is deposited in the GenBank under the accession no. KP412983.
